# Connectomic Analysis of Brain Networks: Novel Techniques and Future Directions

**DOI:** 10.3389/fnana.2016.00110

**Published:** 2016-11-09

**Authors:** J. Leonie Cazemier, Francisco Clascá, Paul H. E. Tiesinga

**Affiliations:** ^1^Department of Neuroinformatics, Donders Institute, Radboud UniversityNijmegen, Netherlands; ^2^Department of Cortical Structure and Function, Netherlands Institute for NeuroscienceAmsterdam, Netherlands; ^3^Department of Anatomy, Histology and Neuroscience, School of Medicine, Autónoma UniversityMadrid, Spain

**Keywords:** connectome mapping, brain clearing, neuronal labeling, whole-brain imaging, mouse connectome, Bayesian modeling, connectome models, Peter's rule

## Abstract

Brain networks, localized or brain-wide, exist only at the cellular level, i.e., between specific pre- and post-synaptic neurons, which are connected through functionally diverse synapses located at specific points of their cell membranes. “Connectomics” is the emerging subfield of neuroanatomy explicitly aimed at elucidating the wiring of brain networks with cellular resolution and a quantified accuracy. Such data are indispensable for realistic modeling of brain circuitry and function. A connectomic analysis, therefore, needs to identify and measure the soma, dendrites, axonal path, and branching patterns together with the synapses and gap junctions of the neurons involved in any given brain circuit or network. However, because of the submicron caliber, 3D complexity, and high packing density of most such structures, as well as the fact that axons frequently extend over long distances to make synapses in remote brain regions, creating connectomic maps is technically challenging and requires multi-scale approaches, Such approaches involve the combination of the most sensitive cell labeling and analysis methods available, as well as the development of new ones able to resolve individual cells and synapses with increasing high-throughput. In this review, we provide an overview of recently introduced high-resolution methods, which researchers wanting to enter the field of connectomics may consider. It includes several molecular labeling tools, some of which specifically label synapses, and covers a number of novel imaging tools such as brain clearing protocols and microscopy approaches. Apart from describing the tools, we also provide an assessment of their qualities. The criteria we use assess the qualities that tools need in order to contribute to deciphering the key levels of circuit organization. We conclude with a brief future outlook for neuroanatomic research, computational methods, and network modeling, where we also point out several outstanding issues like structure–function relations and the complexity of neural models.

## Introduction

Ever since the pioneering work of Ramon y Cajal, the neuron doctrine has shaped our understanding of the brain: neurons are the functional units of the nervous system and their interactions are at the basis of all brain processes. As a result of the marked subcellular polarization of neurons, information in brain networks flows mostly in a single direction: from a dendrite of a cell via its soma to its axon, and from there, through specialized intercellular contacts (synapses) to a second neuron or neurons (Ramón y Cajal, 1909; Bullock, 1959). This paradigm has enabled researchers to perform modeling studies that investigate the properties of some neuronal circuits (e.g., Buszáki, 1989; Schneidman et al., 2006). However, in order to understand and to model the workings of any brain circuit, it is necessary to first identify all its parts, and to quantitatively elucidate how they are interconnected. That is why a growing number of researchers currently aim to create increasingly complete quantitative connection diagrams of specific neuronal networks, and ultimately of all the neurons in an animal (e.g., the Human Brain Project, www.humanbrainproject.eu; or the Mouse Connectome Project, www.mouseconnectome.org). Such partial or complete precise wiring diagrams have been termed a “connectome” (Sporns et al., 2005; Jarrell et al., 2013; Sporns, 2013). Given the massive numbers and morpho-functional diversity of neurons, as well as 3D intricacy and submicron size of their connections, and the fact that axons often extend to remote brain or body regions, the technical challenges involved in tracing connectomes with cellular resolution are staggering (de Costa and Martin, 2013). In fact, the only complete connectome produced to date is that of the small worm *Caenorhabditis elegans*, which has just 302 neurons of 118 morpho-functional types, which form about 5000 synapses between them. By extension, the term “Connectomics” is now applied to the area of neuroanatomy explicitly aimed at elucidating quantitatively, and with increasing cellular resolution, the wiring of brain networks (Emmons, 2015; Shibata et al., 2015).

More specifically, wiring diagrams with cellular resolution are indispensable for testing the assumptions that are used in brain modeling research. Markram et al. (2015) recently combined anatomical and electrophysiological data to create a digital reconstruction of the local microcircuitry in a small piece of rat neocortex. This reconstruction enabled them to perform *in silico* (computer simulation) experiments on how information is processed by the neurons in a neocortical domain. The assumptions that underlie such models need to be tested and refined in order for researchers to be able to create biologically meaningful models. An assumption that is often referred to and that may be tested using connectome data is Peters' Rule (Peters and Feldman, 1976; Mishchenko et al., 2010; Tiesinga et al., 2015), which postulates that the presence of a synapse between an axon and a dendrite can be inferred based on their proximity. More knowledge about the connectome could prove or disprove these and other assumptions, and in this way more insight could be gained on how biologically plausible certain brain models are. For example, results from Kasthuri et al. (2015) suggested that Peters' Rule does not in fact provide sufficient information for inferring synapses. Of course, apart from determining the cell type-specific numbers of neurons and measuring their connections, neural dynamics, and modulation of the exchange of information between neurons will eventually have to be incorporated to connectomic data to predict real brain function and dysfunction (Bargman and Marder, 2013; Deco and Kringelbach, 2014; Wang and Krystal, 2014).

Among the available neuronal labeling methods, the most relevant for connectomic analysis are those able to produce complete axonal and dendritic staining of one or a few neurons. These include the intracellular or juxtacellular labeling with chemical markers such as biocytin or a variety of tagged dextrans (Pinault, 1996; Reiner et al., 2000), as well as transfection with viral vectors that drive the expression of high levels of fluorescent proteins in a few or even single cells (Kuramoto et al., 2009; Wang et al., 2014; Porrero et al., 2016). Alternative approaches rely on the generation of specific transgenic mice lines in whose brains high expression of label proteins is restricted (either by random insertion or through a promotor-specific Cre-lox system) to small neuron populations or a given cell type (Wouterlood et al., 2014; Saunders and Sabatini, 2015; see below).

Given the complexity and intermingling of dendrites and axons of nearby cells, reliable reconstruction and measurement requires a very low density labeling, ideally one or few cells per brain (Kuramoto et al., 2009; Economo et al., 2016). The combination of intracellular recordings with biocytin labeling in *ex vivo* brain slice preparations (~400 μm-thick) has proven especially fruitful. This method has allowed the production of detailed catalogs of virtually all the neuron types in localized regions of juvenile cerebral cortex or hippocampus, based on both their somatodendritic and local axon morphologies and their membrane properties (Li et al., 1994; DeFelipe et al., 2013; Markram et al., 2015). This technique, however, is not ideal for use on adult brain tissue, as adult cells are more vulnerable to damage by the sectioning procedure (Huang and Uusisaari, 2013; see however Mohan et al., 2015, on adult human biopsic samples). In any case, however, labeling in slices is unsuitable to study the vast axonal trees of long-range projection neurons, as they usually extend far beyond the dimensions of any viable slice preparation (Kuramoto et al., 2009; Economo et al., 2016).

Tracing and measuring the dendritic and axonal arborizations of a labeled neuron requires scanning under light microscopy (LM) sized brain volumes, often an entire brain. Three main technical solutions have been proposed: (a) classic histological serial sectioning, mounting and subsequent computer-aided alignment, and 3D tracing; (b) microscope imaging of tissue block surface while sectioning it; or (c) making the brain tissue transparent by means of chemical clearing methods, and then imaging it without sectioning using laser-sheet illumination and long-distance microscope optics. Each methodology presents its own advantages and limitations for a connectomic analysis (reviewed in Osten and Margrie, 2013).

Unambiguously resolving synapses and critical subcellular features in the pre- and post-synaptic elements (such as volume, synaptic active zone area and shape, etc.) requires electron microscopy (EM). Most such measurements require 3D visualization. Precise 3D rendering can be achieved using (a) serial ultrathin sectioning with standard or automated tape-collection and subsequent Transmission EM imaging; or (b) ion-beam milling combined with serial block-face imaging with Scanning EM (FIB-SEM; Bosch et al., 2015; reviewed in Kubota, 2015).

One of the most important challenges that arises when gathering connectome data is combining data from different scales, each of which is measured by different methods (Sporns, 2013; Yook et al., 2013). EM is best suited for imaging at the subcellular/synaptic scale (Helmstaedter et al., 2008). However, local and long-range circuits also need to be investigated at the regional and whole-brain scales, respectively. For such research, LM is more suitable. Combining data from these different scales has proven to be difficult, as the interrogated tissues are often treated (immunostained, sliced, etc.) according to a specific research method, which leaves them unsuitable for re-analysis by other methods that may measure at a different scale.

Nevertheless, the achievable imaging resolution of LM is improving, and new strategies are being used to identify certain structures when using EM (Osten and Margrie, 2013). For example, electron microscopic 3D methods are particularly informative when combined with cell-selective dextran or transfection tracing (Anderson et al., 2009; Suzuki et al., 2015). Likewise, when using correlative light and electron microscopy (CLEM), tissues used for LM may be further processed for EM or vice versa (De Boer et al., 2015). Such methods require careful handling of the tissue with specific strategies, as regular LM and EM preparations are often mutually exclusive (De Boer et al., 2015). Furthermore, various labeling and tissue clearing methods like mammalian GFP reconstitution across synaptic partners (mGRASP) and clear, lipid-exchanged, anatomically rigid, imaging/immunostaining compatible, tissue hydrogel (CLARITY) have recently been developed, which make cell type-specific labeling and imaging of at least some neuronal structures possible in unsectioned brains (Kim et al., 2012; Chung et al., 2013). These are only a few examples of the tools that are currently being developed with the goal of mapping mammalian brain networks with cellular resolution.

Most of the techniques mentioned above have been recently reviewed (Reiner et al., 2000; Lanciego and Wouterlood, 2011; Mitra et al., 2013; Wouterlood et al., 2014; Osten and Margrie, 2013; Kubota, 2015; Nassi et al., 2015; Susaki and Ueda, 2016; Treweek and Gradinaru, 2016), and thus will be dealt only briefly here. Interested readers are referred to these reviews for further details. In addition, some new tools and methods, which are often a combination and refinement of the above ones, have been introduced in the past few years which may turn to be useful for connectomic studies of the mouse brain. In the following sections, we will focus on these recent methods. In addition, their merits and known limitations for connectomic analysis will be compared and evaluated: for a given research question, what method or data would be best to use and why? These overviews and evaluations may be particularly helpful for researchers who are entering connectome research and are gathering information on the diversity of experimental methods in this field. Finally, we will give a short future outlook on labeling and imaging tools and discuss how data from different experiments can be combined computationally.

## Criteria for evaluating experimental methods

In order to provide a comparison of the experimental methods we describe, we first need to define relevant criteria to compare. The overview tables provide information on these criteria for each research method, and can hence be consulted when deciding on which methods to use for any specific research goal.

### Resolution of labeling and imaging

When selecting a method, it is of critical importance to consider whether the available methods allow you to measure the desired structures at the desired scale. An important factor that contributes to this criterion is the type of microscopy used. While only EM (typically working at 4000X–20,000X magnification) can resolve synaptic structures, such detailed imaging and the corresponding data analysis currently can cover only a minute part of the circuit, typically containing no more than a thousand synapses. As EM is time- and labor-intensive it is currently not feasible to map large brain networks based on EM data (Helmstaedter et al., 2008). In contrast, LM works in the 40X–1000X magnification range and thus allows you to gather data within the context of the larger tissue. LM may allow visualizing a typical mammalian neuron's dendritic tree and long axonal arbors in its entirety provided that they are well-labeled (see below), but its resolution is not sufficient to ascertain the presence of synapses. Even super-resolution fluorescence microscopy (with a resolution of a few tens of nanometers) might not have the resolution necessary for determining complete neuronal wiring diagrams (Huang et al., 2010). There are currently many different types of LM that can be used, each with their own (dis)advantages (Osten and Margrie, 2013).

### Properties of labeling strategies

**Figure 1:**
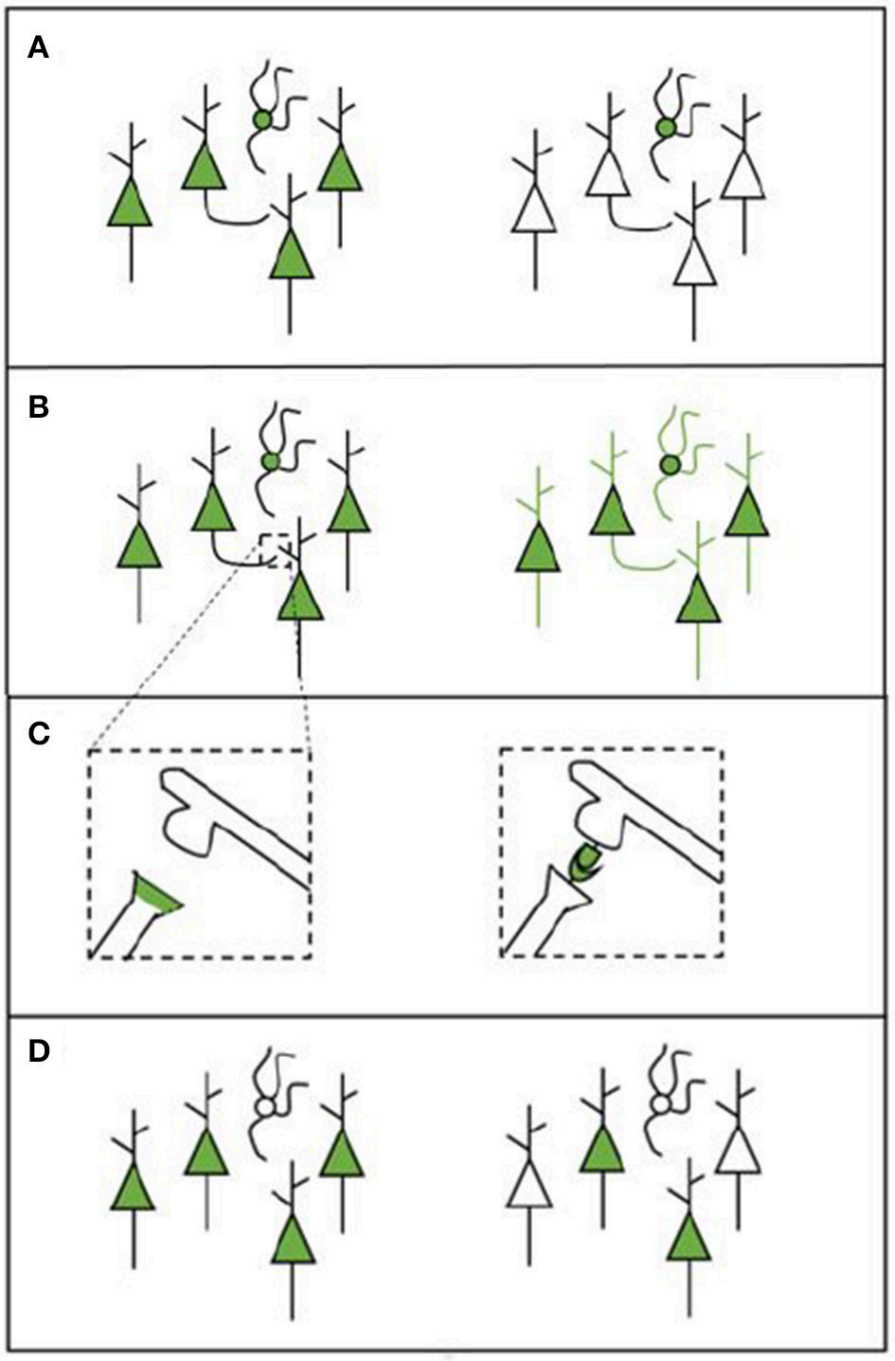
**Schematic overview of various labeling strategies. (A)** A labeling tool may label all cells in the region of choice (left) or only cells that belong to a specific cell type (right). **(B)** A labeling tool may label only certain parts of a cell (e.g., the soma, left) or it can label the entire cell in order to enable projection tracing (right). **(C)** Some experimental procedures enable synapse labeling. We provide two examples here: labeling synapses by labeling proteins that are present in the pre-synaptic terminal (left); or to create a chemical reaction in the synaptic cleft using components from both the pre- and the post-synaptic neuron (this is called transsynaptic labeling, right). The transsynaptic strategy depicted here is mGRASP. **(D)** The labeling that is performed can be broad (left) or sparse (right), depending on how many cells are labeled.

Apart from the type of microscopy system used, there are a number of other factors that define whether the desired structures can be measured (see Figure 1). The experimental protocol will also influence what type of data is gathered and how this can be analyzed. Each of the following factors should be considered when designing a labeling strategy.

- Cell-type specificity: Some tools are able to target only specific cell types (e.g., a cell type that expresses a particular gene or cells that innervate a particular target). This enables researchers to gain information on the locations, numbers, and synaptic partners of certain types of cells (see Figure 1A).
- Whole-cell morphology: Some techniques make it possible to image the morphology of the entire cell. This enables gathering data across scales, which is necessary if the goal is to trace projections (e.g., Yuan et al., 2015). For projection tracing, both an adequate labeling strategy and microscopy strategy are needed (see Figure 1B).
- Synapses: The possibility to identify the presence of a synapse (and when using EM, the synapse type) enables researchers to gather information about the connections that are present in the network. Some tools may label synapses by labeling synaptic proteins or by creating chemical reactions in the synaptic cleft (see Figure 1C). Other labels enable the inference of synapses by transsynaptically labeling neurons that are upstream or downstream from a given neuron or neuron population (Yook et al., 2013).
- Coverage: Apart from deciding upon a type of labeling, it is important to consider the coverage of the various possible tools. Different tools can label different amounts of cells in one single experiment. Bulk-labeling methods, even under the best conditions, reveal only the average connections of large neuron populations; since these methods lack cellular resolution, they are of limited use for quantitative connectomics. Bulk-labeling techniques must thus be complemented with single-cell labeling methods to quantitatively resolve the wiring diagram of a given neuronal type. Single-cell labeling methods, however, are still time and labor intensive (see Figure 1D).

### Ease of use of experimental methods

When two methods can yield similar results, then the easiest method would be preferred. Various qualities of research methods can contribute to this. Firstly, several different animal species can be used and those animals can be manipulated in ways that are more or less time- and labor-intensive. For example, *in utero* electroporation of mouse embryos, which is performed in mGRASP (Druckmann et al., 2014), is a difficult and low-consistency procedure to use compared to methods that use commercially available transgenic mouse lines that consistently express neuronal labeling proteins in specific neuronal populations. Furthermore, the time- and labor-intensiveness of the experimental procedure and its success rate are factors that need to be considered, as well as the time required for imaging and data processing. Finally, research methods that use sectioned brains are required to achieve the best resolution (both with LM and EM), but have the disadvantage of sectioning distortions, with subsequent alignment difficulties. Methods that use and image whole intact brains may therefore be preferred when lack of cellular or subcellular resolution is not an issue (Ertürk et al., 2012). It must be noted that the success rates of the experiments described are not always mentioned in the available literature. These will therefore not be included in this review.

### Reliability of the acquired data

With the previously described requirements, we can assess whether a tool can provide information of sufficient quality about a sufficient number of cells and with how much ease this can be done. Although this is the core of the information that we need for mapping brain networks, there are some other issues that need to be taken into consideration. When performing any type of research, the data obtained need to be reliable; the tool needs to measure what it is supposed to measure. Proper validation of the used acquisition methods is thus an essential component. Other qualities that may be assessed are the ability to image live brains or the ability to perform multiple rounds of tissue staining so that multiple cell groups can be analyzed in a single brain.

### Data analysis

For any type of experiment to be successful the appropriate analysis tools should be available. While modern technology is making the gathering of great amounts high-resolution data easier, these data would be of no use if it cannot be processed appropriately. Currently, along with simple and consistent single-cell labeling (Porrero et al., 2016) data analysis is in fact one of the two key limiting steps in connectome research (Helmstaedter, 2013): no automated projection tracing software is available that outperforms human annotators and hence most projection tracing is performed manually (but see also http://diademchallenge.org, where several algorithms can be found that were the result of an automated projection tracing-competition). Mapping all the myriad of highly diverse mouse brain neuronal networks and synapses with manual tracing may take thousands or even millions of hours. Smart solutions for data analysis are therefore highly necessary (Helmstaedter, 2013). Nevertheless, in the meantime, substantial progress can be achieved from the intelligent application of human effort into fully resolving a number of key model networks at the cellular and subcellular level. The second current limiting step is the combination of different types of information from a great amount of experiments will be essential. Hence, the current challenge is the creation of software pipelines required for such analysis.

## Contemporary neuroanatomic research methods

Here, we will first describe and evaluate several molecular tools that are currently used in connectome research. Then, we will continue to do the same for a number of different imaging tools, which include brain clearing strategies and new microscopy approaches.

### Molecular tools

A number of molecular tools have recently been developed that can aid research on the connectome. Some specifically aim to reveal synapses, whereas other methods are more focused on, for example, unraveling the trajectories of single neurons throughout the brain. We will start out by describing tools for synapse labeling, after which we will describe methods that enable other types of molecular labeling for connectome research. An overview of all the discussed molecular methods and their qualities is provided in Table 1.

**Table 1:** **Overview of molecular tools for researching the connectome**.

**Name**	**Imaging technique^a^**	**Cell type specific**	**Whole cell morphology^b^**	**Imaging synapses^c^**	**Animal model**	**Manipulation**	**Duration experiment^d^**	**Imaging intact brain**	**Other^e^**	**Reference**
GESEM	1	+	−	1	Mouse or Drosophila	Transgenic animals, injections	5 weeks	−	−	Atasoy et al., 2014
STaR	2, 3	+	−	1	Drosophila	Transgenic animals	8 days	+	1	Chen et al., 2014
mGRASP	2	+	+	1	Mouse	In utero electroporation and injections	11 weeks	−	−	Kim et al., 2012; Druckmann et al., 2014
iBLINC	4	+	−	1	*C. elegans*	Transgenic animals	1 day	+	1, 2	Desbois et al., 2015
Transsynaptic viral tracers ^f^	Various types of LM	+	+	2	Mouse	Various	Several days up to several weeks	−	−	Lo and Anderson, 2011; Ginger et al., 2013
Double co-injection tract tracing	2	−	+	3	Mouse	Injections	2 weeks	−	−	Zingg et al., 2014
Bulk injection of viral tracers	5	−	+	3	Mouse	Injections	4 weeks	−	−	Oh et al., 2014
iDISCO	3, 6	+	0	0	Mouse	Transgenic animals	3 weeks	+	−	Renier et al., 2014
BROPA	7	−	−	1	Mouse	−	7 weeks	−	−	Mikula and Denk, 2015

a *As used in the indicated literature. 1, TEM; 2, confocal laser scanning microscopy; 3, 2-photon microscopy; 4, compound fluorescence microscopy; 5, STP; 6, light sheet ultramicroscopy; 7, serial block-face EM*.

b *+, the research tool meets this criterion with adequate resolution and/or coverage to inform computational methods; 0, not enough information in literature*.

c *1, synapses can be localized; 2, synapses can be inferred by transsynaptic transport of label; 3, no information about synapses; 0, not enough information in literature*.

d *Including preparations like IUE or viral injections, not including the creation of transgenic animals or viral vectors. Also not including imaging and data processing times as these were often not mentioned in literature*.

e *1, imaging in live animals; 2, multiple rounds of staining possible*.

f *These qualities depend on the type of type of virus that is used and other experimental conditions. Here, we do not review 1 type of viral labeling but whether the criterion can be met using a viral label (e.g., do cell-type specific viral labels exist?)*.

### Tools for labeling synapses

**Figure 2:**
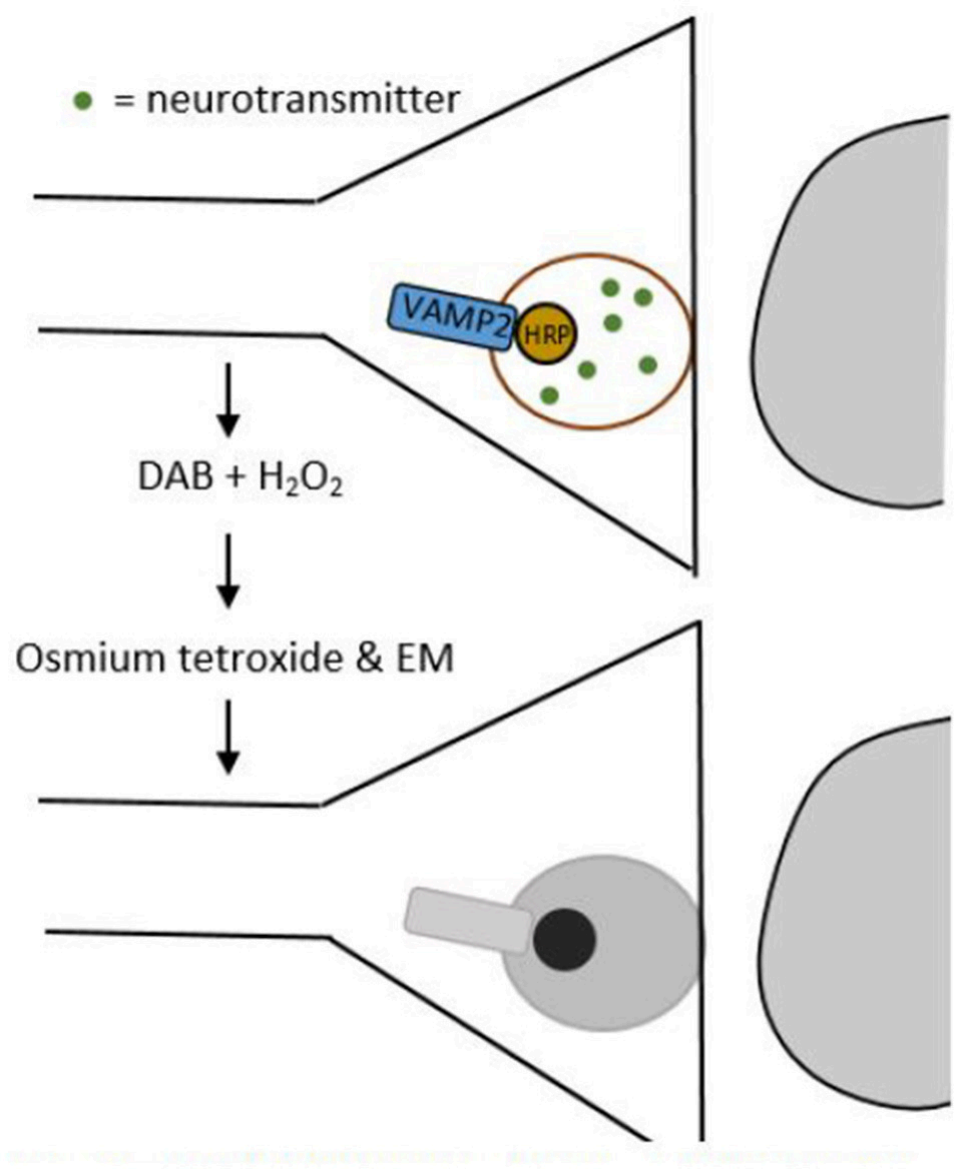
**Schematic drawing of a GESEM experiment**. First, horse radish peroxidase (HRP) is added to the synaptic vesicle by tethering it to the vesicle associated membrane protein 2 (VAMP2). This step can be made cell-type specific by using a recombinase system like Cre/LoxP. Then, the tissue is treated with 3,3′-diaminobenzidine (DAB) and hydrogen peroxide (H_2_O_2_), which generates a polymeric precipitate. When this precipitate is treated with osmium tetroxide, it becomes electron dense, which makes it appear as a dark area through an electron microscope.

In EM images synapses are identified through their ultrastructural details like post-synaptic densities and synaptic vesicles in the pre-synaptic cell. However, since EM can only image small pieces of tissue that do not contain entire neurons, we need smart approaches to unravel which cell types are connected by the synapses imaged (Helmstaedter et al., 2008). Atasoy et al. (2014) provided an elegant solution to this issue with their genetically encoded synaptic marker for electron microscopy (GESEM) (Figure 2).

GESEM works as follows: first, horse radish peroxidase (HRP) is tethered to the vesicle associated membrane protein 2 (VAMP2). This creates a VAMP2:HRP complex that locates HRP inside the synaptic vesicle. In order to make the expression of this complex cell-type specific, the VAMP2:HRP coding sequence is placed in a Cre recombinase (Cre)-dependent recombinant adeno-associated virus (rAAV) targeting vector. By injecting this Cre-dependent VAMP2:HRP vector into transgenic mice that only express Cre in specific cell types, VAMP2:HRP becomes expressed selectively in only those cell types that transgenically express Cre (see Box 1 and/or Huang and Zeng, 2013, for a more detailed explanation of genetic manipulation with recombinase systems). The HRP in the synaptic vesicle can then be visualized by creating a polymeric precipitate using 3,3′-diaminobenzidine (DAB) and hydrogen peroxide. Processing this precipitate with osmium tetroxide makes it electron dense, which increases electron scattering in transmission EM (TEM) and makes it appear as a dark area on the image. The dark areas in the image thus correspond to synaptic vesicles, permitting the identification of axonal release sites of specific cell types using EM. Atasoy et al. (2014) used this tool to express VAMP2:HRP in synaptic vesicles of Agouti-related peptide (AGRP) and proopiomelanocortin (POMC) neurons, which helped them characterize the features of the axonal projections of these cell types.

Box 1 **Using recombinase systems for genetic manipulation**

**Figure 3:**
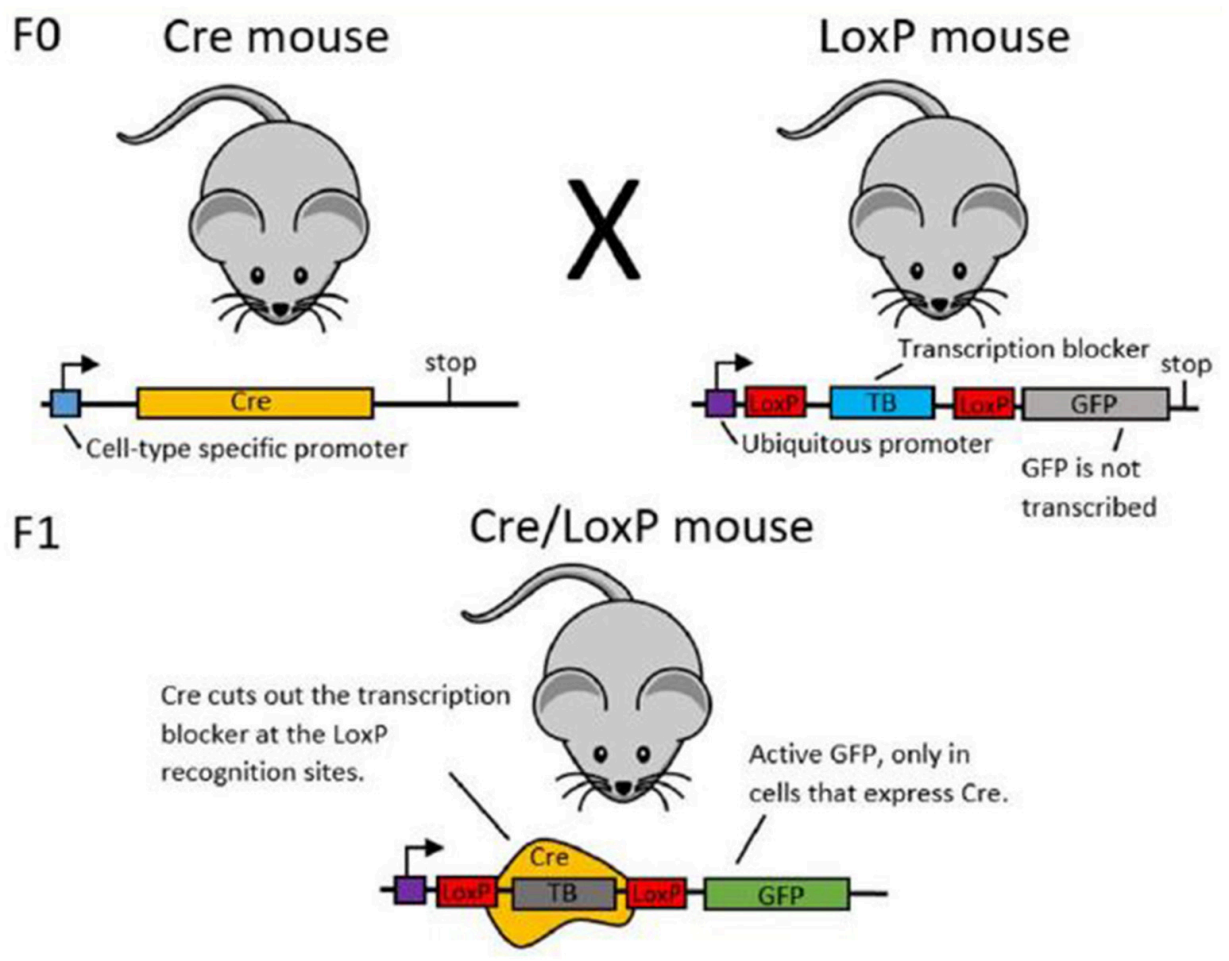
**Schematic illustration of the Cre/LoxP system**. In the F0 generation, mouse line 1 **(left)** expresses Cre under a cell-type specific promoter. Mouse line 2 **(right)** expresses the labeling protein (here: GFP), but has an upstream transcription blocker, which prevents transcription of GFP. When these two mouse lines are crossed, some offspring will have both the Cre DNA and the LoxP-TB-LoxP-GFP DNA. In these animals, Cre is expressed only in the desired cell type and in this cell type, Cre cuts out the transcription blocker at the LoxP sites. This enables GFP transcription and thereby cell-type specific labeling.

In order to establish cell-type specific expression of transgenes, researchers may use recombinase systems. Here, we use the Cre/LoxP system as an example for explaining the function of recombinase systems (see Figure 3). Green Fluorescent Protein (GFP) is the example transgenic protein to be expressed.

To establish GFP expression in only one type of cell, first two transgenic mouse lines are created (see Gama Sosa et al. (2010) for more information about the creation of transgenic animals). The mice in line 1 have the DNA code for the Cre recombinase protein under the promoter of a different protein. If Cre is under the promoter of a protein that is only expressed in a certain cell type, Cre will also only be expressed in this particular type of cell. The mice in line 2 transgenically express the desired cell-type specific transgenic protein (here: GFP). Upstream of the GFP, there is a stop sequence which ensures that GFP will not be transcribed and hence not expressed. This stop sequence is flanked by two LoxP sites.

When you cross mouse lines 1 and 2, some offspring will express both the transgenic Cre site and the transgenic LoxP-GFP site. In these animals, Cre recombinase will cut out the stop sequence upstream of the GFP at the LoxP sites. With the stop sequence now removed, GFP will be expressed. As Cre recombinase was only expressed in a certain cell type, GFP will also only be expressed in that specific cell type.

A similar option for cell-type specific transgene expression is to use only 1 mouse line, which expresses Cre, and to then inject a Cre-dependent protein into these animals (as is used in Atasoy et al., 2014). There are a number of different recombinase systems analogous to the Cre/LoxP system, for example the FLP/FRT system and the R/RS system (both used in Chen et al., 2014).

GESEM may actually be useful for identifying the synaptic terminals of a small and/or scattered neuronal population projecting into a given area. But for most cases, as far as the cells of origin of a pathway are confined to an area or nucleus, reliable and highly specific labeling and EM visualization of synaptic terminals can be far more readily achieved with conventional anterograde BDA or PHA-L tracing (Wouterlood and Jorritsma-Byham, 1993).

Concurrent with this EM approach, some new approaches for identifying synapses using LM were also recently developed. One of those methods is called synaptic tagging with recombination (STaR) (Chen et al., 2014). STaR is similar to some other synaptic labeling techniques in that it tags synaptic proteins in order to visualize synapses. An improvement that is achieved in STaR is that the tagged synaptic proteins are still expressed under their endogenous regulatory elements. This prevents over-expression or inappropriately located (non-endogenous) expression of the measured synaptic proteins (Chen et al., 2014).

For STaR, which has so far only been performed in the visual system of *Drosophila melanogaster*, both a pre- and a post-synaptic marker were designed. The pre-synaptic marker was based on Bruchpilot (BRP), a protein that is expressed pre-synaptically in *Drosophila*. The endogenous BRP was modified in a bacterial artificial chromosome so that it included a flippase recognition target (FRT) stop-sequence and a tag downstream of this sequence. The tag can enable immunostaining of BRP, or can be a fluorescent protein (FP) itself. The modified BRP was introduced in FLP transgenic flies (see Box 1 for a more detailed explanation of genetic manipulation using recombinase systems). When the FRT stop-sequence is excised by FLP recombinase, the synaptic tag is expressed, but only in those neurons that transgenically express FLP (Golic and Lindquist, 1989). A similar inducible system was created in order to tag the histamine-gated chloride channel 2 (Ort) for post-synaptic labeling.

After both pre- and post-synaptic labels were tested separately, Chen et al. (2014) decided to simultaneously label pre- and post-synaptic sites in partner neurons. In this experiment, they used the FLP system for Ort labeling and therefore needed a different inducible system for cell-type specific BRP labeling in the same animal. They opted for the R Recombinase inducible system, which induces cell-type specific gene expression in a way similar to that of the FLP system (Nern et al., 2011). Although these inducible systems are similar, they do not show a cross-reaction so that the labeling performed by the two systems remains cell-type specific (Nern et al., 2011). After the simultaneous pre- and post-synaptic labeling overlapping synaptic puncta could be imaged, indicating reliable synaptic labeling in *Drosophila* for *in vivo* imaging.

An important aspect to note in STaR, is that it uses the physical proximity of labeled pre- and post-synaptic proteins to infer the locations of synapses. Another way to infer such physical proximity or even a physical synaptic contact, is by ensuring that labeling requires an interaction between pre- and post-synaptic structures. This is called transsynaptic labeling. Two tools that use this strategy are mGRASP (Feng et al., 2012; Kim et al., 2012; Druckmann et al., 2014) and iBLINC (Desbois et al., 2015).

Non-mammalian GFP reconstitution across synaptic partners (GRASP) was first developed for *C. elegans* by Feinberg et al. in 2008, but has since been extended for use in the mouse brain (mGRASP: Kim et al., 2012). In mGRASP, a green fluorescent protein (GFP) is split into two fragments. These two fragments are both brought to the synapse: one fragment is tethered to the pre-synaptic protein neurexin-1ß, the other fragment is tethered to the post-synaptic protein neuroligin-1. Only if these fragments are in close proximity to each other, the GFP will be reconstituted and be fluorescent (see Figure 1C). Each complex itself also includes a fluorescent protein; mCerulean for the pre-synaptic complex and dTomato for the post-synaptic complex. These can be used for examining the locations of the individual pre- and post-synaptic complexes.

Furthermore, the genetic code for each of the complexes may contain inducible elements like Cre/LoxP (see Box 1 for a more detailed explanation of genetic manipulation using recombinase systems). For performing selective labeling, first Cre recombinase is introduced in (a specific region of) the mouse brain. This can be done using *in utero* electroporation. After this, Cre-dependent mGRASP complexes can be introduced using a viral vector, leading to cell-type or area-specific synaptic labeling. An example of what can be done using this technique has been published by Druckmann et al. (2014), who used Cre-dependent mGRASP to label the synapses of hippocampal CA3-CA1 projections.

*In vivo* biotin labeling of intercellular contacts (iBLINC) is a new technique that has only been used in *C. elegans*. Instead of using a transsynaptic reconstitution like mGRASP, which is permanent, iBLINC uses a transsynaptic enzymatic reaction. The product of this enzymatic reaction can be visualized using LM, but may also be turned over for a new round of staining, which makes iBLINC a useful dynamic imaging tool for use *in vivo* (Desbois et al., 2015). In order to create an enzymatic reaction in the synapse, the biotin ligase enzyme BirA is built into the neurexin-1 (NRX-1) protein (creating a BirA-NRX-1 complex) and an acceptor peptide (AP) is built into the neuroligin-1 (NLG-1) protein (creating an AP-NLG-1 complex). In Desbois et al. (2015), specific promoters are used to transgenically express the BirA-NRX-1 and AP-NLG-1 complexes in pre-synaptic AFD sensory neurons and post-synaptic AIY interneurons, respectively. Other types of neurons may also be targeted. The modified pre- and post-synaptic proteins enable the enzymatic attachment of biotin to the post-synaptic acceptor protein by the pre-synaptic biotin ligase enzyme. The AP-biotin complex can subsequently be labeled by streptavidin with red fluorescent protein (RFP) tethered to it, expressed by transgenic scavenger cells called coelomocytes. This way Desbois et al. (2015) were able to image puncta that correspond to one or a small number of synapses.

Table 1 summarizes the qualities of GESEM, STaR, mGRASP, and iBLINC. Firstly, it must be noted that STaR and iBLINC have yet to be adapted for mammalian research. Since the mouse is the animal of choice for many connectome studies, GESEM and mGRASP currently seem to be more relevant synaptic labeling tools for connectome studies. GESEM and mGRASP have some qualities in common as they both provide cell type specific labeling of synapses and take several weeks to months to perform. However, since GESEM is a tool for EM and EM reconstruction of neuronal projections and connections is very time- and labor-intensive (Kasthuri et al., 2015), using mGRASP currently seems to be the better option for mapping larger neural networks. In Chen et al. (2014) the issue of non-endogenous development of synapses is raised, but mGRASP likely avoids such issues, as Kim et al. (2012) have shown that mGRASP does not affect synaptogenesis.

If we turn our attention to the non-mammalian synaptic labeling techniques, they also have specific advantages and disadvantages with implications for their future use. Firstly, STaR can currently provide images with better resolution than iBLINC. Also, whereas most other transgenic synaptic labeling techniques introduce a transgene under non-endogenous regulatory elements like modified promoters, STaR is performed differently. In STaR, only the coded proteins are modified and not their regulatory elements, so that non-endogenous expression of the proteins is avoided (Chen et al., 2014). The creators of iBLINC do not mention performing experiments that control for non-endogenous expression. They do state that the iBLINC labeling resembled that of GRASP, which was shown to label real synapses without altering synaptogenesis (Kim et al., 2012; Desbois et al., 2015). An advantage of iBLINC is that it is very dynamic, since the product of the enzymatic labeling reaction can be turned over to enable new rounds of labeling. Also, the label can be altered by photo-convertible fluorescence to compare various labeling rounds more easily. If these qualities could be extended to mammalian brains, perhaps combining them with advanced imaging techniques, this could improve our insight into the dynamics of synaptic connectivity between specific types of neurons.

An important caveat of the above techniques is that, in the best scenario, they allow visualizing the presence and spatial distribution of synapses between two cell populations that are previously known to be connected, as genetic modifications need to be selectively introduced beforehand in the pre- and post-synaptic elements of the circuit. For this reason, beyond relatively simple and accessible invertebrate nervous systems, these techniques remain at present more a proof of principle than an efficient tool for mammalian brain studies. Moreover, except the GESEM technique, the other methods rely on LM, and thus remain qualitative in nature and blind to key synaptic parameters such as the size of the synaptic active zone, mitochondrial content, and vesicle pool size and distribution.

### Other molecular tools for studying brain anatomy

Apart from tools that specifically label synapses, there are a variety of other tools that may help researchers to reach their goal of reconstructing a mammalian connectome. Here, we will describe and evaluate a number of such molecular tools. For an overview of the tools discussed and their qualities, see Table 1.

### Using viruses for neuronal labeling

It has been known for decades that certain virus strains are capable of trans-neuronal transport (see for example Card et al., 1990). The viruses used so far for this purpose are the rabies virus, the herpes simplex-virus and lentiviruses. Currently, a variety of modified virus strains are being used that express fluorescent proteins in the infected cells (Wickersham et al., 2007; Beier et al., 2011; Lo and Anderson, 2011; Ginger et al., 2013; Schwarz et al., 2015). These viruses may infect cells retro- or anterogradely; this directionality is thought to be determined by the type of glycoprotein expressed in the viral envelope (Beier et al., 2011). Furthermore, viruses need to replicate themselves after infection of a cell before they can infect a next cell. Therefore, when polysynaptic marker viruses are made to be replication-incompetent, they cease to transport themselves across multiple synapses and can be used as monosynaptic transsynaptic markers (Beier et al., 2011). It is also possible to ensure that the virus only infects specific cell types. For such experiments transgenic animals with inducible systems like Cre are used and sometimes also helper viruses. Helper viruses may for example make transgenic Cre expressing cells susceptible for the labeling virus (Wall et al., 2010; Beier et al., 2011).

Such adaptations of viral vectors facilitate tracing up- and downstream pathways of specific cell types. Schwarz et al. (2015) showed how these methods can be combined with advanced imaging methods: in their experiment, monosynaptic projections onto a cell population in the lateral entorhinal cortex of the mouse brain were labeled using a modified rabies virus. Subsequently, they cleared the mouse brains and imaged them using light-sheet fluorescence microscopy. Light sheet fluorescence microscopy is different from regular fluorescence microscopy in that the fluorescence excitation is performed horizontally (from the side of the specimen) while the image is captured from above, in the vertical plane (see also **Figure 5**). This enables fast imaging without mechanical sectioning and prevents out-of-focus bleaching (Osten and Margrie, 2013; Schwarz et al., 2015). The combination of all these relatively new techniques led to the acquisition of images that could be used to trace the labeled neuronal projections.

Though the field of viral neuronal labeling is still progressing, some disadvantages have to be named. First of all, viruses, especially the rabies virus, can be hazardous to laboratory staff, so precautions need to be taken when working with them (Kelly and Strick, 2000). The toxicity of viruses is also an issue; infected tissues die a number of days after performing the injections (Yook et al., 2013). Furthermore, virally-mediated transfections are difficult to direct to a specific location, because viral particles remain viable for hours after injection, and have a tendency to spread through the intercellular brain space. Viral vectors also have variable infective capacity and promoter potency (the ability to induce the expression of the transgene protein products). In some cases, the viral vectors (e.g., retroviruses) can only infect mitotic neuronal precursors and thus can only be applied on embryonic brains. An alternative for directing the virus to a specific location is to inject or electroporate the vector RNA or DNA construct directly into the neuron *in vivo* (Porrero et al., 2016).

A final issue is the effectiveness of the transfection. Some authors have reported false negatives where not all the neurons connected to the target area may be labeled (Yook et al., 2013). Reversely, Beier et al. (2011) performed electrophysiological recordings to test the reliability of their transsynaptic viral label and reported synaptic currents in only 5/8 labeled cells. This indicated that three of the eight labeled connections may have been false positives, although they did provide some alternative explanations for why they did not record synaptic currents in these cells. If a transsynaptic viral label could be injected into a single cell, the neurons projecting to it could be identified for that specific targeted cell. For example, Marshel et al. (2011), succeeded in labeling monosynaptic inputs to single cells by using two-photon microscopy guided electroporation. Their success rates for *in vitro* and *in vivo* experiments were 12 and 35%, respectively, and in the *in vivo* experiments each successfully electroporated single neuron yielded an average of 49 labeled monosynaptic inputs. But given these low success rates and the high sampling requirements of connectome research, such a protocol would not be very suitable for connectome research in practice: a very large amount of experiments would need to be performed to effectively map local networks.

**Figure 4:**
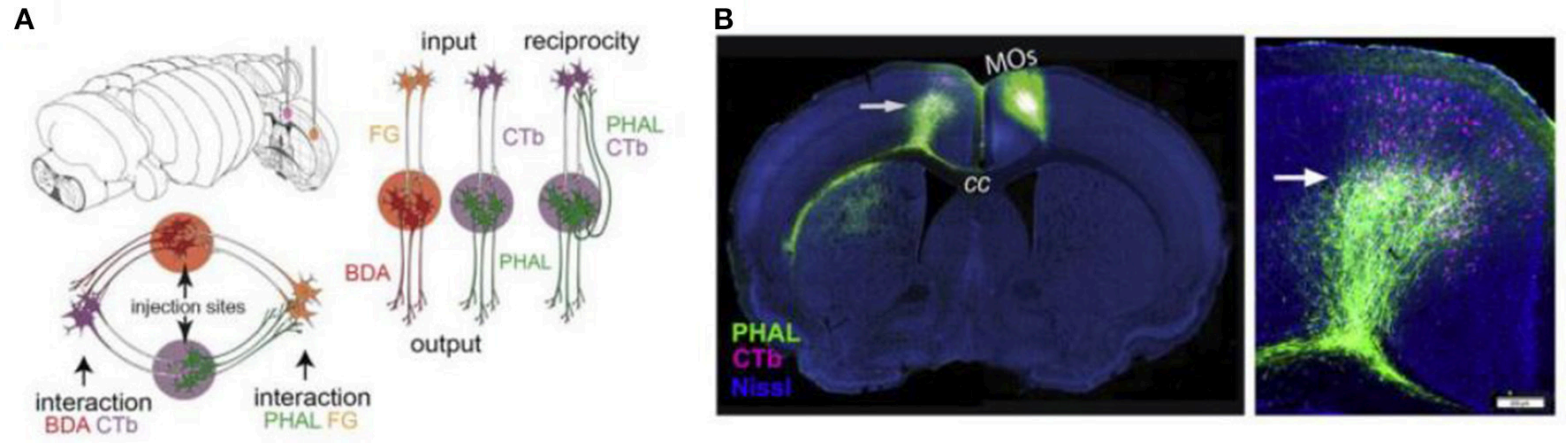
**Projection tracing using double co-injection labeling. (A)** Schematic illustration of how four different types of pathways can be labeled using four different tracers in two injection sites. PHAL, *Phaseolus vulgaris* leucoagglutinin; BDA, biotinylated dextran amine; CTb, cholera toxin subunit b; FG, Fluorogold. **(B)** Example fluorescence images showing one injection into a secondary somatomotor area (Mos) (left) and an area both providing input (cells labeled in pink) and receiving output (cells labeled in green) from this area in the opposite hemisphere (right). Adapted from Zingg et al. (2014, p. 1098). Copyright 2014 by Elsevier Inc.

A viral approach that has been used for connectome research is double co-injection viral tracing. In this and other tracing experiments, we have to keep in mind that purely unidirectional transport does not seem achievable: most tracers transport bidirectionally, but transport in one direction can strongly prevail (Lanciego and Wouterlood, 2011). In double co-injection tracing, adult wild-type (WT) mice are injected with tracers in two non-overlapping locations. Each location receives two different tracers: one anterograde tracer and one retrograde tracer. The anterograde tracers label axons that arise from the co-injection sites and their terminals, while the retrograde tracers label upstream neurons that innervate the co-injection sites. In total, four different tracers are used and each of these tracers has its own distinctive fluorescent protein. This way of labeling allows the researchers to label four types of connections: (1) inputs to the co-injection sites; (2), outputs from the co-injection sites; (3) recurrent connections, which are labeled by both the antero- and retrograde tracer from one specific injection site; and (4) intermediate stations, since if an area is labeled by tracers from both sites of co-injection it is an intermediate station between these two regions (see Figure 4) (Zingg et al., 2014). After performing many injections of tracers in different locations and imaging the results using laser scanning microscopy, Zingg et al. (2014) acquired data from over 600 labeled pathways. Using this data, they created a cortico-cortical connectivity map of the mouse brain and concluded that the entire cortex is organized into several sub-networks that can interact through select intermediate cortical areas (see also the Mouse Connectome Project, www.mouseconnectome.org).

A similar project is that of the Allen Mouse Brain Connectivity Atlas (see also http://connectivity.brain-map.org/). Recently, this project reported on creating a brain-wide, cellular-level, mesoscale connectome for the mouse brain (Oh et al., 2014). The strategy used for this project is slightly different from that used for the Mouse Connectome Project. In the Allen Mouse Brain Connectivity Atlas project, the authors injected rAAVs expressing EGFP in specific regions of the brains of WT mice. These viral vectors labeled all the efferent axonal projections from the injection site. Just like in the Mouse Connectome project, a great number of brain areas were injected and traced. The results were imaged using serial two-photon tomography, an imaging tool discussed later in this review. The resulting open-access mesoscale connectome helped to create a wiring diagram for several cortical regions and thalamic nuclei (Hunnicutt et al., 2014; Oh et al., 2014).

Even though the efforts of Oh et al. (2014) and Zingg et al. (2014) have provided researchers in the field of connectomics with a lot of useful information, these approaches do also have some downsides. Firstly, these methods do not label synapses, therefore, after tracing the projections from an injection site, it is not possible to assess where the labeled cells transmit their information. Secondly, the two approaches use bulk injections instead of cell type-specific labeling. These factors make that reconstructing neural networks based on this information would not be feasible without making certain predictions or assumptions about the missing information. Oh et al. are therefore working on what they call Phase II of their project: mapping the projections of genetically defined neuronal populations, using Cre transgenic mice and Cre-dependent viral vectors.

### Non-viral neuronal labeling methods

Double co-injection tracing and bulk viral injections are both performed by imaging sections of the labeled mouse brain. During the past few years, new brain clearing techniques have enabled whole-brain imaging, which prevents alignment difficulties that may occur when imaging sections. A labeling technique that was designed specifically for labeling brains in combination with a clearing technique is iDISCO (immunolabeling-enabled three-dimensional imaging of solvent-cleared organs) (Renier et al., 2014). iDISCO is designed to be used with the clearing technique 3DISCO, which is discussed later in this review (Ertürk et al., 2012). In iDISCO, mouse brains (which may be transgenic) are labeled using immunohistochemistry: the brain is incubated in a solution including antibodies that can recognize the protein that is to be labeled (these are called primary antibodies). Some primary antibodies have a FP attached to them. If this is not the case, a second incubation needs to be performed with antibodies that recognize the primary antibody and label it using a FP (these are called secondary antibodies). It must be noted that not all types of antibodies are compatible with iDISCO (see Renier et al., 2014). After the protein labeling steps, the brain is cleared using 3DISCO. This type of clearing involves incubations in several concentrations of tetrahydrofuran and subsequent incubations in dichloromethane and dibenzyl ether. Using different types of (transgenic) animals and immunolabels, this versatile approach can label various structures in a cell type-specific manner and enable whole-brain imaging of these tissues (Renier et al., 2014).

Whole-brain labeling tools also exist for combination with electron microscopy. Though whole-brain EM is not feasible yet, preparing the brain in its entirety before EM sectioning makes the sections more similar, which makes it easier to align the sections and trace projections. A tool that facilitates this is BROPA (brain-wide reduced-osmium staining with pyrogallol-mediated amplification) (Mikula and Denk, 2015). BROPA is based on conventional ROTO EM staining methods (Willingham and Rutherford, 1984), but yields a number of improvements. When the reduced-osmium-incubation includes formamide, the staining can reach much deeper into the mouse brain and hence is more uniform. Furthermore, thiocarbohydrazide can be replaced by pyrogallol for reduced bubble formation in the tissue (Mikula and Denk, 2015). The authors state that BROPA-prepared brains appear to meet the requirements for performing neural circuit reconstructions of the mouse brain. However, as stated before, given the submicron scale of brain synapses and staggering 3D complexity of brain microcircuitry, EM reconstructions of the entire mouse brain may remain an unattainable goal for a long time.

## Imaging tools

Here, we will describe several recently developed imaging tools that are being used in connectome research. First, we will address clearing protocols, which make the brain transparent to enable whole-brain imaging with light microscopy. Then, we will continue to discuss a number of advanced microscopy approaches that can aid connectome research. Tables 2, 3 provide an overview of the described clearing methods and microscopy tools, respectively.

**Table 2:** **Overview of tissue clearing methods**.

**Technique**	**Type of clearing**	**Experiment time^a^**	**Degree of clearing^b^**	**Microscopy^c^**	**Immuno-staining**	**FP emission**	**Imaging synapses^d^**	**Whole-cell morphology^d^**	**Other^e^**	**Reference**
Sca*l*e	Hyperhydration	Weeks to months	+	1, 2	−	+	−	+	1, 2, 3, 6	Hama et al., 2011
CUBIC	Hyperhydration	2−3 weeks	+	3	+	+	+	+	2, 3, 4, 6	Susaki et al., 2014
3DISCO	Solvent-based	4 days	+	1, 3	+	+/−	0	+	5, 6	Ertürk et al., 2012, 2014
SeeDB	Simple immersion	1 week	−	1	−	+	−	+	−	Ke et al., 2013
ClearT2	Simple immersion	1 day	−	Various	+	+	−	−	7	Kuwajima et al., 2013
CLARITY	Hydrogel embedding	2−5 weeks	+	2, 3, 4	+	+	+	+	6, 8	Chung et al., 2013

a *Time needed for clearing of an entire mouse brain. Not including imaging and data processing times as these were often not mentioned in literature. Also not including preparations like creating transgenic animals*.

b *+, sufficient for whole-brain imaging; −, limited*.

c *As used in the indicated literature. 1, two-photon microscopy; 2, confocal laser scanning microscopy; 3, light-sheet microscopy; 4, single-photon microscopy*.

d *+, the research tool meets this criterion with adequate resolution and/or coverage to inform computational methods; 0, not enough information*.

e *1, fragile tissue after clearing; 2, tissue expansion; 3, loss of proteins; 4, compatible with small primate brains; 5, tissue shrinkage; 6, loss of lipids; 7, can be used with lipophilic dyes; 8, relatively expensive*.

**Table 3:** **Overview of novel microscopy approaches**.

**Technique**	**Imaging time^a^**	**Data processing**	**Section thickness**	**Resolution^a^**	**Whole cell morphology^b^**	**Imaging synapses^b^**	**Other^c^**	**Reference**
STP	24 h	- Automated image registration. - Computer-aided stitching and warping. - Informatics Data Pipeline developed for Allen Brain Atlas	100 μm	~ 1 μm per pixel	+	+	–	Ragan et al., 2012
(2p)-fMOST	9 days	- Automated image pre-processing - Manual segmentation of brain areas - Manual reconstruction of neurons - Computer-aided transformation to brain atlas	2 μm (optical sections)	~0.5 μm per pixel	+	+	–	Gong et al., 2013; Zheng et al., 2013
CLEM	Varies	Varies	Varies	~1 μm and several nm per pixel	+	+	1, 2	De Boer et al., 2015

a *Imaging times are for imaging an entire mouse brain at the highest possible resolution for the technique. Lower resolutions may be faster. Not including labeling experiments or data processing*.

b *+, the research tool meets this criterion with adequate resolution and/or coverage to inform computational methods; 0, not enough information*.

c *1, very specific tissue treatments required for LM/EM transfer; 2, LM tissue cannot be stored*.

### Clearing protocols

In order to understand how clearing methods work, we first need to understand how tissues become opaque. This is explained in detail in Richardson and Lichtman (2015). In short, when a traveling light wave approaches an atom, some of its energy may be transferred to an electron of the atom. If the amount of transferred energy (the photon) is insufficient for the electron to jump to a different orbital of the atom (which would cause fluorescence), the electron will vibrate shortly, thereby releasing the energy in the form of a new light wave. There are two important differences between the old and the new light wave. Firstly, while the old light wave was moving in only one direction, the new light wave is sent in all directions: the light is scattered. Secondly, the brief interaction with the electron causes a temporary pause in the movement of the light wave: the light is slowed down for a few femtoseconds. This delay of the light propagation is the basis of what is called the refractive index (RI) of the medium, which is a measure that describes how fast light propagates through a specific material relative to vacuum (Hecht, 2002). Tissues with different molecular densities cause different amounts of delay in the light propagation and hence each tissue has its own refractive index.

Now, what is it that makes tissues transparent or oblique? Transparent materials like air or water have a very uniform density of scattering molecules. This uniformity ensures that light only coherently propagates through the material along the direction of the original light wave (see Richardson and Lichtman, 2015, on how laterally scattered light waves are canceled out). In materials like biological tissue however, a lot of different molecules are present (proteins, lipids, etc.) that act as scatterers. These molecules are packed into membranes and other structures that are non-uniformly distributed similar to raisins in raisin bread. This non-uniform distribution makes the light scatter in such a way that laterally scattered light is not canceled out, hence biological tissues are opaque. From this we can conclude that, in order to make tissues transparent, the inhomogeneity of the light scattering by membranes needs to be resolved. This is referred to as making the RI of the tissue more uniform. Again, this is only a brief summary of the physics that underlie tissue transparency. For a more detailed explanation, see Richardson and Lichtman (2015).

There are various methods that can be used to make the RI of a tissue more uniform, and these methods can be grouped into four different approaches. We will describe each approach and provide examples of clearing protocols that are used regularly (see Richardson and Lichtman (2015) for a more extensive review on tissue clearing). Table 2 gives an overview of all the protocols described here and their qualities. As brain clearing tools are often not limited to a specific molecular labeling method, we will not address all the same qualities as we did for the labeling tools. Brain clearing-specific factors that will be discussed are the degree of clearing, fluorescent protein emission and the possibility to perform immunohistochemistry experiments combined with clearing.

Over the past few years, various clearing methods have been created and improved for brain research. Sca*l*e was one of the first of those (Hama et al., 2011). Sca*l*e uses the principle of hyper-hydration. In this approach, the brain is incubated in an aqueous solution that contains a detergent that removes the lipids from the brain. As lipids have high refractory indexes, this step reduces the RI of the tissue. The clearing solution also contains a substance that will hydrate hydrophobic regions of high refractive index proteins, again decreasing the tissue's RI (Richardson and Lichtman, 2015). In Sca*l*e, the lipid detergent used is Triton X-100 and the hydration is caused by urea in glycerol. The incubations last for several weeks up to months, and leave the tissue with a RI of ~1.38, matching that of the Sca*l*e solution (Hama et al., 2011). The transparency of Sca*l*e-treated tissues is sufficient for whole-brain imaging, but Sca*l*e has a number of downsides. First of all, lipid membranes are the structural and functional substrate of brain circuits, thus the Sca*l*e procedure dissolves precisely the most relevant elements from the tissue, leaving behind just a protein scaffold. Moreover, as a result the tissue becomes very fragile during the clearing procedure. Also, Sca*l*e clearing cannot be combined with immunohistochemistry experiments. These factors, combined with the long experiment duration, make that Sca*l*e was quickly replaced by other clearing protocols.

A newer clearing protocol that also uses the hyper-hydration strategy is CUBIC (clear unobstructed brain imaging cocktails and computational analysis) (Susaki et al., 2014). In CUBIC, the brain is first incubated in a mixture of high Triton X-100, urea and an amino-alcohol that improves brain tissue solubility so that the lipids are removed within 1 week. The tissue is then washed in phosphate-buffered saline (PBS) to prepare it for a second incubation. The second incubation solution includes Triton X-100, urea and an amino-alcohol, which makes the RI of the tissue more uniform, also in regions deeper in the brain (Susaki et al., 2014). Just like Sca*l*e, CUBIC enables whole-brain imaging. Still, CUBIC outperforms Sca*l*e as it can be combined with immunohistochemistry and can perform clearing much faster than Sca*l*e. A downside of the CUBIC procedure is that the incubation in high concentrations of Triton X-100 not only removes lipids but also causes loss of up to 41% of protein content (Chung et al., 2013). This can affect immunohistochemistry since such experiments rely on protein labeling, so CUBIC may not be the ideal protocol to combine with immunohistochemistry. An interesting aspect of CUBIC is that it can clear tissues larger than mouse brains, providing the opportunity to not only image whole rodent brains but even primate brains such as that of the marmoset (Susaki et al., 2014).

Another approach to clear tissues is solvent-based clearing. This involves one or more incubations in a solvent that dehydrates the tissue and solvates or removes lipids. This makes the tissue more homogeneous and dense, thus increasing the RI. The high RI tissue then needs one or more subsequent incubations in a solution that matches this RI and may solvate additional lipids. A complicating factor is that dehydration removes water molecules that are necessary for fluorescent protein emission (Richardson and Lichtman, 2015). Ertürk et al. (2012) solved this issue in their 3DISCO protocol. 3DISCO involves a number of incubations in increasing concentrations of tetrahydrofuran (THF) for dehydration and lipid solvation and subsequent incubations in dichloromethane and dibenzyl ether for additional RI-matching. The fluorescent protein emission after this protocol is sufficient for imaging, but after about 2 days the fluorescence disappears (Ertürk et al., 2012). However, if iDISCO-compatible dehydration-resistant dyes are used, iDISCO immunostaining can replace imaging of FPs (Richardson and Lichtman, 2015). 3DISCO has already been shown to enable axonal tracing and imaging of spines (Ertürk and Bradke, 2013; Ertürk et al., 2014).

The third approach that may be used to clear tissues is simple immersion. This approach makes for simple experimental procedures, as it consists of placing the tissue in a solution of a high RI molecule and letting it clear in this solution for a certain amount of time. Generally, the high RI molecule needs a refractory index >1.45 to clear the hydrated samples (Richardson and Lichtman, 2015). One of the clearing reagents that can be used for this approach is See Deep Brain (SeeDB), which consists of a saturated solution of fructose in water with 0.5% α-thioglycerol (Ke et al., 2013). Because the RI of SeeDB is close to that of lipids, it enables clearing of both gray and white matter. Furthermore, while many other clearing protocols affect the tissue size, SeeDB does not. Since its development SeeDB has been used for various experiments; e.g., for research on the peripheral nervous system of mouse embryos (Genç et al., 2015) and research on human eyes (Bergua et al., 2015). Since the penetration of SeeDB is limited, SeeDB does not effectively clear very large tissues. Also the clearing of whole mouse brains using SeeDB has been shown to take substantially more time than that of smaller samples like hemi-brains (Ke et al., 2013).

Around the same time that SeeDB was developed, Kuwajima et al. (2013) developed ClearT2. ClearT2 is a mixture of formamide and polyethylene glycol in water. It can clear tissues considerably faster than SeeDB since only one day of clearing is needed. Also, ClearT2 can be used in combination with immunohistochemistry, so for most experiments ClearT2 would be preferred over SeeDB. ClearT2 has the same limitations as SeeDB in that the penetration of the clearing agent is limited so mature mouse brains cannot be made fully transparent. Kuwajima et al. (2013) also developed a similar clearing agent called ClearT, which provides better transparency than ClearT2. However, ClearT is only compatible with lipophilic dyes (e.g., cholera toxin subunit B), and not with immunostaining or built-in fluorescence proteins. So as was argued in the section on molecular tools, the specific research goal should be leading in the choice for a specific experimental protocol.

The last and most complicated approach to clearing is hydrogel embedding. In this approach, hydrogel monomers are introduced into the tissue and these are subsequently cross-linked to embed the entire tissue in a polymer hydrogel. After this embedding step, the lipids can be removed from the sample without protein loss as the proteins are captured within the hydrogel mesh. A final incubation for optimal refractive index matching makes the tissue ready for imaging (Richardson and Lichtman, 2015). An effective protocol that uses this approach is CLARITY (clear, lipid-exchanged, anatomically rigid, imaging/immunostaining compatible, tissue hydrogel; Chung et al., 2013). In CLARITY, acrylamide and bisacrylamide are the monomers of choice, which are introduced in the tissue together with formaldehyde and thermally triggered cross-linking initiators. Polymerization is initiated by incubating the tissue at 37°C. The tissue subsequently undergoes electrophoretic tissue clearing, which uses the electrical charge of the lipids to extract them from the tissue. The final step of RI-matching can be performed by immersing the tissue in a solution of 85% glycerol or FocusClear, which was developed specifically for CLARITY (Chung et al., 2013). Though CLARITY is not the fastest or simplest method for brain clearing, it has the advantage that CLARITY-treated brains can be labeled by immunohistochemistry, even with multiple rounds of staining and washing. Given the fact that protein loss is minimal, CLARITY is probably one of the best approaches to use when combining brain clearing with immunolabeling. Furthermore, Tomer et al. (2014) developed COLM (CLARITY optimized light sheet microscopy), which makes it possible to clear, label and image an entire mouse brain within 1 month. Another addition was proposed by Yang et al. (2014), who introduced PACT (passive clarity technique) for improved clearing of various tissue types, even bone, without electrophoresis (see also Treweek et al., 2015). CLARITY has been used for clearing both mouse (e.g. Spence et al., 2014) and human (e.g., Ando et al., 2014) brain samples, but also to clear other mouse organs like liver, lung, and kidney (Lee et al., 2014).

### Advances in microscopy

**Figure 5:**
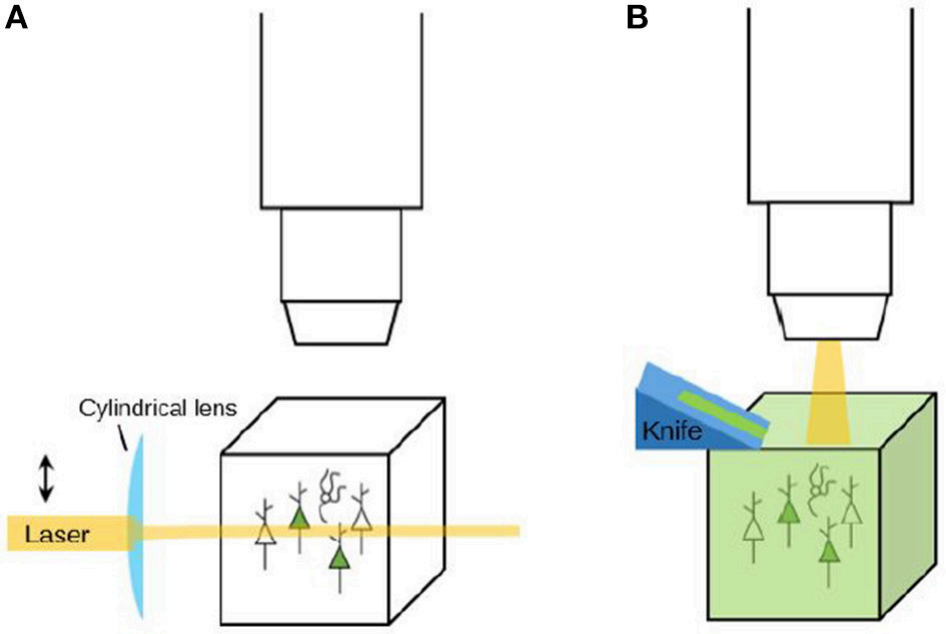
**Imaging strategies: clearing and optical sectioning vs. physical sectioning (tomography). (A)** When a labeled tissue is cleared, there is no need to cut the tissue. Instead, tools like light-sheet microscopy (as depicted here) can be used to selectively illuminate the tissue and thereby perform *optical sectioning*. **(B)** When a tissue is not cleared, it needs to be sectioned in order to reveal the labeling that is present in deeper layers. A tomography system is a system that automatically sections and images a tissue. Sectioning may be performed before imaging (the resulting tissue ribbons can then subsequently be imaged) or after imaging (to reveal a new layer of tissue for imaging).

After labeling and/or clearing a tissue, the experiment is not finished yet: the tissue still needs to be imaged. Experimental tools may provide researchers with brains with detailed labeling, but to extract useful data from these brains, microscopes are needed that can image them at a high resolution within an appropriate amount of time. Although the combination of speed and resolution is a tough one to realize, several recent advances in microscopy that provide solutions for this will be described here (see Table 3 for an overview). All three microscopy tools reviewed here are tomography systems: they slice the brain to enable imaging of deeper layers. When the used brain tissue is cleared, there is no need for tomography since the transparent tissue can be optically sectioned using for instance light sheet microscopy (see Figure 5). Knowledge of both these strategies is needed to make informed decisions on imaging methodology.

In 2012, Ragan et al. developed serial two-photon tomography (STP), a method that combines automated vibratome sectioning of tissue with automated two-photon microscopy. Various approaches may be used to fluorescently label cells in the brain before imaging with STP. Then, after the mice are anesthetized and perfused, the brains are dissected and embedded in agarose for hardening of the tissue. The STP procedure itself can be performed by an integrated microscope like a TissueCyte 1000 (TissueVision, Somerville, MA, USA). During imaging, the tissue is fixed to a motorized stage. For each layer of tissue, two-photon tomography with femtosecond lasers is used to take a Z-stack of optical sections of 0.5 μm of thickness at a depth of about 50 μm under the tissue block surface. After imaging of the optical sections, the top 50 μm of the tissue is sectioned off by the vibratome, and the imaging of a new stack of optical sections can begin (Ragan et al., 2012). This method in principle could enable 3D imaging of entire brains at 0.5 μm in-slice resolution within a week of 24 h/day automated work was also used by Oh et al. (2014) to create their mesoscale mouse connectome. An advantage of the STP-strategy of deep imaging and subsequent slicing is that abnormalities in the field of view due to sectioning difficulties are reduced (Yuan et al., 2015). Though STP provides the possibility of high-resolution 3D imaging, to date only 50 μm spaced serial section of mouse brains have been published (Allen Brain Mouse Connectome Atlas) and no single-cell 3D projection tracing experiments using STP have been performed. Therefore, before STP can be used broadly for connectomics research, proof-of-principle experiments of high-resolution projection tracing should first be performed (Amato et al., 2016).

Another set of recently developed methods is that of several types of micro-optical sectioning tomography systems (MOST) (Li et al., 2010; Gong et al., 2013; Zheng et al., 2013). In a MOST system, a resin-embedded tissue is mounted in a chamber that can move in all directions. The motorized chamber repeatedly pulls the tissue past a knife, thereby creating ribbons of tissue of about 30 μm thick. The tissue ribbons can then be imaged immediately after sectioning, while still in motion (Li et al., 2010). The short working distance and section thinness allows for maximal-resolution light microscopy. However, a complicating factor in this set-up is that plastic embedding generally reduces fluorescence in a tissue. Gong et al. (2013) provided a solution for this issue with their improved glycol methacrylate embedding that is optimized for fluorescent protein-labeled mouse brains. Furthermore, they designed an opto-acoustical deflector that increases the imaging speed of the MOST system by improving the system stability for long imaging times (Gong et al., 2013; Yuan et al., 2015). Using this new system, named fMOST, Gong et al. (2013) not only confirmed a number of previously discovered pathways but also identified several previously unreported putative projection pathways in the mouse brain.

An additional improvement was achieved when Zheng et al. (2013) developed 2p-fMOST. This technique uses two-photon imaging and combines the embedding protocol and the improved imaging speed of fMOST with the tissue sectioning strategy of STP. After embedding, the tissue is mounted in the motorized chamber, which navigates the tissue so that a first ribbon can be imaged beneath the surface. After imaging, the ribbon is cut with the knife and the next layer of tissue is exposed for imaging. Using 2p-fMOST, a whole mouse brain can be imaged in 9 days at a resolution that is suitable for axonal projection tracing (Zheng et al., 2013). So to summarize, while 2p-fMOST imaging is considerably slower than STP, it does provide the necessary resolution for connectomics research.

While imaging the brain with methods like STP or MOST can provide detailed information on brain cell morphology, additional information may be needed, necessitating even higher resolution imaging. For example, assessing synapse types by their morphology can at present only be performed using EM. What makes the combination of LM and EM difficult is that very high-resolution LM is needed for performing adequate image overlay. Additionally, brain preparation procedures for EM and LM imaging are often mutually exclusive, i.e., LM preparations may alter the ultrastructural characteristics of a tissue. These issues are gradually being overcome by ongoing developments in the field of correlated light and electron microscopy (CLEM; De Boer et al., 2015). An example of how CLEM may be used in neurobiology research is provided by Urwyler et al. (2015). They performed LM on labeled *Drosophila* mechanosensory neurons, and subsequently validated their synaptic labeling method using EM analysis. For correlating the different types of microscopy, they used near-infrared laser branding (NIRB): during LM, the tissue was marked by slightly damaging the area around the region of interest with a laser, which makes it recognizable for subsequent EM imaging (Bishop et al., 2011). Though CLEM is still in development and it is not used very widely at present, it could prove to become a powerful tool to connect the various levels of connectome research in the near future.

## Future directions in neuroanatomic research methods

We will now proceed to discuss how some of the reviewed techniques and possible combinations of them could inform connectome research. Since double co-injection tracing has a very large coverage, it is a tool that can aid researchers to gain an understanding of the mesoscale network connections in certain brain areas or in the entire brain. This is exactly what Zingg et al. (2014) used the technique for. However, this strategy does not label neurons in a sparse manner; nor does it label specific cell types. Due to this, the amount of information that can be gathered about individual cells is limited. In order to be able to create more detailed models of brain networks, experimental protocols are needed that can inform scientists about the location of specific cell types, their synaptic partners and the locations of the synapses.

Druckmann et al. (2014) have already shown that mGRASP is an adequate tool to use when studying cell-type specific synaptic connections in the mouse hippocampus. However, since the methods used by Druckmann et al. (2014) comprise sparse labeling of synapses (from ca. 50 to 200 cells), using such methods it would take a large number of experiments to image all the connections in a large network like that of the hippocampus. When using large numbers of animals for network mapping, individual variation can impede the research process. Furthermore, performing a large amount of mGRASP experiments for connectome research would only be useful if a validated additive data processing pipeline were available. These issues are both caused by the relatively low coverage of mGRASP. This coverage is already being improved upon; MacPherson et al. (2015) developed an activity-dependent mGRASP protocol for Drosophila, which uses multiple inducible systems for labeling the active synapses of multiple cell types with different fluorescent proteins. If this protocol could be extended to mammalian research, that would decrease the number of experiments needed for network reconstructions based on mGRASP.

iDISCO is easier to perform than mGRASP, as it does not involve the difficult procedure of in utero electroporation. It is also relatively fast and inexpensive, and the used immunohistochemistry approach is very versatile, so many different cell types could be labeled (Renier et al., 2014). However, iDISCO has not been applied for projection tracing experiments very often thus far. As the authors are still focusing on improving 3DISCO (Ertürk, 2015), projection tracing might be one of the future possibilities when using this clearing method. If 3DISCO/iDISCO could be used for projection tracing and synapse labeling experiments, it would be a relatively quick and versatile method to use for performing them.

In contrast to iDISCO, CLARITY has been used for both projection tracing experiments and labeling synapses (Chung et al., 2013). A disadvantage of CLARITY is that it is considerably more expensive than iDISCO. Also, the experimental procedure is slightly more complicated. Hence, when starting up a new research project that uses brain clearing, it would be important to first weigh the advantages and disadvantages of CLARITY, iDISCO and other possible clearing methods.

2p-fMOST also seems like a very suitable tool for gathering connectome data. The authors have already succeeded in long-range projection tracing (Gong et al., 2013), but they have not yet used 2p-fMOST for imaging synapses. They did show that 2p-fMOST has a resolution sufficient to image spines (Zheng et al., 2013). Imaging synapses using specific labels therefore does seem to be within the range of possibilities when performing 2p-fMOST experiments.

In parallel with the development of new clearing protocols and imaging devices, an urgent area for progress is developing methods for consistently labeling the entire axonal tree of functionally identified neurons with high sensitivity and signal/noise ratio. Imaging devices, no matter how sophisticated, will only achieve cellular resolution when dendrites, somas and axons have been sparsely, completely and specifically labeled (Porrero et al., 2016).

Apart from further development of the research tools mentioned above, there are several other new research methods that might prove to be useful for neuroanatomical research. Firstly, iBLINC and STaR might be adapted for mammalian use and thereby provide additional methods for synaptic labeling in mouse brains. Also, a group of researchers recently presented a new inducible system that will enable sparse labeling of specific cell types. This system works with two interleaved pairs of lox sites with a relatively large but variable distance in between them. Because Cre will focus its activity toward those lox sites that are the least far apart, the FP is then only expressed in about 10% of the cells that express Cre (Ibrahim et al., 2015). Additionally, He et al. (2016) recently showed that combinations of different targeting strategies (e.g., genetic targeting, viral targeting, and fate mapping), can be used to effectively target restricted GABAergic subpopulations. This leads the way for the interrogation of other cell types using combinatorial techniques.

In 2012, Zador et al. proposed how connectivity analysis could be performed using cellular RNA bar codes as labels instead of fluorophores. And indeed, very recently they presented MAPseq (Kebschull et al., 2016). In MAPseq, each neuron in a specific region is infected with a virus that expresses a unique RNA bar code created by a recombinase. These viruses are capable of intracellular transport, and whenever the virus travels through a cell to its projecting location, the RNA bar code travels with it. After the infection and transport, the projection pathways of individual cells can be identified by sequencing specific pieces of brain tissue. Kebschull et al. (2016) used this technique to quantify the amount of projections of single locus coeruleus cells and the locations of these projections. An important advantage of this approach is that the number of available bar codes outnumber the available fluorophores for individual labeling of cells. The approach thus has a very large dynamic range (Zador et al., 2012; Pollock et al., 2014; Kebschull et al., 2016). Moreover, this procedure leaves out image acquisition and segmentation, and instead uses RNA sequencing as main analysis strategy. This might actually be a very fruitful strategy, as image segmentation is still not fully automated and hence expensive to perform, while sequencing technology is well-developed and relatively cheap (Marblestone et al., 2014).

Some of the techniques described here might be combined for use in connectomics research. The various clearing methods described in this review may be combined with a number of different labeling techniques, although not all possible combinations can be made and each combination of labeling and clearing will have its own strengths and weaknesses. For example, since CLARITY involves the least protein loss out of the clearing tools described here, CLARITY is probably one of the best approaches to use when combining immunolabeling of proteins with brain clearing (Chung et al., 2013). However, CLARITY renders the tissue useless for subsequent electron microscope analysis of synapses. This is just one example of the considerations that should be made when designing an experiment. Hence, sufficient knowledge of the available labeling and clearing protocols is needed for creating state-of-the-art experiments.

Yuan et al. (2015) suggest that fMOST might be combined with newly developed tracing methods in order to accelerate the characterization of information flow in brain circuits. A possible combination is that of mGRASP labeling and 2p-fMOST imaging. 2p-fMOST has thus far not been used with synaptic labeling, and mGRASP could potentially be an approach to prepare mouse brains for detecting actual synapses. Vice versa, the level of coverage of mGRASP has been limited in the experiments performed until now. 2p-fMOST imaging could improve the coverage of mGRASP by taking away the need for sectioning the brain and imaging the separate sections. The possibility of imaging the entire brain at a high resolution within a short amount of time could implicate that sparse labeling in mGRASP could be replaced by broad labeling. This would enable brain mapping based on smaller numbers of animals than was previously conceived.

Another approach that needs to be mentioned here is CLEM. Although a lot of CLEM approaches are still being developed and optimized, some CLEM experiments have been performed that show its potential for future use (e.g., Urwyler et al., 2015). It would not be advisable to combine CLEM with CLARITY, iDISCO or various other clearing methods because of the lipid- and protein loss that often occurs in these methods. The corresponding ultrastructural changes would make the tissue unsuitable for EM analysis (Richardson and Lichtman, 2015). On the other hand, various labeling tools like mGRASP or viral labels might be imaged using EM after LM analysis. The EM analysis could validate the used labeling tools and additionally aid in the identification of various synapse types (Atasoy et al., 2014; Urwyler et al., 2015). In summary, when combining a labeling method with imaging tools, one will have to opt for either combining labeling with brain clearing and subsequent high-resolution LM imaging, or with CLEM, as CLEM and brain clearing are generally mutually exclusive.

## Computational methods

The anatomical methods reviewed in the preceding text will not provide a complete connectivity necessary for building a detailed simulation of a complete brain. For instance, because they have incomplete coverage, insufficient resolution or can only provide information about a small part of the circuit. The information extracted from a particular experiment will need to be combined with other experiments with the same technique focused on the same brain area, but in different subjects and with other experiments using a different modality.

The general framework in which this integration can be achieved is Bayesian modeling and data fusion, for which recently some progress has been made using human neuroimaging data (Hinne et al., 2014, 2015). This can serve as a guide to achieving similar goals for connectivity at the cellular level. In these studies, data was obtained from two sources: diffusion tensor imaging, which yielded streamlines from one brain area to another, and functional imaging, which yielded temporal correlations between the activity of two different brain areas. Each of these sources provides information regarding the presence of a connection between brain area A and B. The goal is to provide an estimate for whether there is a connection and how strong that connection is. The Bayesian analysis is based on a generative model for the measured data based on the presence and strength of the connection. The properties of this connection are given by another generative model characterized by unknown parameters, which are drawn from a distribution characterized by hyperparameters. Taken together, the generative model is used to calculate the likelihood for the measured data, given specific values for the unknown parameters of the connectivity. The key of the Bayesian model is to invert this likelihood and turn it into a probability distribution for these unknown parameters. In Box 2 we provide more information on this method. This general method makes it possible to combine many relevant measurements using different subjects and modalities into an estimate of the connectivity.

Box 2 **An example of Bayesian modeling**

Bayesian analysis is the ideal tool to incorporate data from different experimental modalities into a unified estimate of a connection probability or an estimate of the associated synaptic properties.

The analysis is based on Bayes' rule. Let n be the presence of a connection, that is, *n* = 1 when there is a connection and *n* = 0 when there is not. Any measurement x that is sensitive to the presence of a connection will have a distribution that depends on the value of *n*, hence we will have a *p*(*x*|*n*). We can either measure this conditional distribution using ground truth data or put in a reasonable estimate based on a measurement model. What we want to know is *p*(*n*|*x*), the probability of a connection *n* = 1 given the value of the measurement *x*. This probability is obtained using Bayes' rule: *p* (*n*|*x*) = *p*(*x*|*n*)*p*(*n*)/*p*(*x*) where *p*(*x*) = ∫ *dn p*(*x*|*n*)*p(n)*. Note that the integral sign here is to be interpreted as an ordinary sum for variables that take discrete variables, such as *n*. You will need an expression for *p*(*n*) to calculate this integral (see below).

When you are making two independent measurements *x* and *y* from the same brain, hence the underlying n can be assumed to be the same, we have both *p*(*x*|*n*) and *p*(*y*|*n*) and these can be combined into a joint distribution *p(x,y,n)* = *p*(*x*|*n*)*p(y|n)p(n)* that we can use in Bayes' rule to get the posterior probability *p*(*n*|*x,y*) = *p*(*x*|*n*)*p*(*y*|*n*)*p(n)*/*p(x,y)* where *p*(*x,y*) = ∫ *dnp*(*x*|*n*)*p*(*y*|*n*) *p(n)*. In this expression *x* and *y* can be completely different types of measurements, for instance, from viral tracing and a DTI measurement on the same brain, or can be the same measurement on multiple occasions, say, two consecutive DTI scans; for the formalism this makes no difference.

It is possible to use only experimental data to estimate the presence of the connection (*n*), but sometimes there also is a prior expectation of what *n* could be, it is then specified by *p*(*n*| θ) and depends on a number of hyper parameters θ specifying this distribution. This prior may of course just reflect the absence of any knowledge, in which case it is flat.

## Discussion

In this review, we have described a large number of novel experimental tools that may inform the field of connectomics. The experimental tools included synapse labeling tools and other molecular labeling tools, as well as imaging approaches like brain clearing protocols and advances in microscopy. Throughout the review we have also discussed the qualities of the described experimental tools, based on several criteria like ease of use and the resolution of the available data.

It must be noted that this review does not discuss every available tool for connectome research. We only described rather recent tools that were developed over the past ~5 years and additionally some older tools that are still being used. Furthermore, we did not include every novel tool in each of the categories, as some tools that were developed <5 years ago are already being outperformed by other tools. For more extensive reviews on neuroanatomical tracing, tissue clearing methods, mapping mammalian synaptic connectivity, novel LM imaging techniques, and brain-wide optical tomography techniques, see Lanciego and Wouterlood (2011), Richardson and Lichtman (2015), Yook et al. (2013), Eberle et al. (2015), and Yuan et al. (2015), respectively. Though we did not review older research methods here, we must note that it is important to not immediately discard the older approaches. Tools that were developed in the past could in the future be combined with newer methods. For example, it has been known for about a century that some materials can clear animal tissues, but this knowledge only became useful when in recent years it was combined with modern brain labeling techniques (Richardson and Lichtman, 2015).

In the next section, we aim to provide a short future outlook with regard to anatomical brain research and brain modeling, while also discussing some outstanding issues for the field of connectomics.

### Recent connectome modeling and reconstructions

Some of the neuroanatomical research methods discussed above have already provided relevant data for brain network reconstructions. For example, Gong et al. (2013) not only confirmed previously discovered pathways while using fMOST, but also documented the discovery of several unreported, putative projection pathways in the mouse brain. Additionally, several mesoscale connectomes have been created based on experimental data. Zingg et al. (2014) used double co-injection tracing to create a cortico-cortical connectivity matrix that helped them identify 8 sub-networks in the mouse brain. Oh et al. (2014) performed hundreds of experiments using (EGFP)-expressing adeno-associated viral vectors to trace axonal projections from defined brain regions. This enabled them to develop the Allen Mouse Brain Connectivity Atlas, an open-access mesoscale connectome of the mouse brain. Although these results have provided new insights in the mouse connectome at the mesoscale level, neither of them measured single neurons or actual synapses, so the connectivity at the microscopic level remained undetected.

If we look into the connectome reconstruction at the microscopic level, we also note some recently published advances. Kasthuri et al. (2015) used EM to image 2250 29-nm coronal slices of a mouse brain, each section with a surface area of 1 mm^2^. From this piece of tissue, they imaged an ~80,000 μm3 box (40 × 40 × 50 μm^3^) at high resolution (3 nm/pixel). Though it took them several years, they were able to create a detailed reconstruction of the piece of tissue based on these images. The reconstruction included labeled axons, dendrites, glia and many sub-cellular components like synapses, synaptic vesicles and mitochondria. This information enabled them to investigate the properties of these components. Their results included information that is relevant for research on brain anatomy and modeling. For example, they concluded that each spine is closely opposed by ~9 different axons, out of which generally only one established a synapse. This has obvious implications for labeling methods that use solely the proximity of neurons to infer synapses (Kasthuri et al., 2015).

While Oh et al. (2014) and Zingg et al. (2014) investigated the connectome on the mesoscale level and Kasthuri et al. (2015) did so on the highest resolution available but in a limited volume, what efforts are being made at the level in between, that of whole single neurons and their synaptic partners? Very recently, Markram et al. (2015) published a paper describing a reconstruction of a rat brain neocortical column. This model was based on experimental research and probabilistic modeling of various characteristics of the column and its components, as was suggested by DeFelipe (2015). In total, it comprises over 30,000 neurons, which have about 8 million connections through ~36 million synapses. The model included experimental data on cell types, cell positions, cell morphologies, synaptic connectivity, synapse types, ion channel types, and electrophysiological properties like conductances and post-synaptic potentials. This enormous reconstruction combines knowledge from many different levels of the connectome and enables *in silico* experiments to reproduce neural activity patterns observed *in vitro* and *in vivo*.

### Unresolved issues in the field of connectomics

At present, a large variety of research tools and connectome modeling strategies are being used in the field of connectomics, each yielding different types of information. However, researchers do not always agree on what the best strategies are for neuroanatomical data collection, analysis and subsequent network modeling. Here, we aim to point out a number of outstanding issues and questions that are relevant for connectomics research.

#### What types of information do we need for connectome reconstructions?

As described in the introduction, when creating connectome reconstructions, the general aim is to better understand brain function and thereby also brain dysfunction. This knowledge could aid researchers in the development of treatments for brain diseases. However, a lot of the research is being performed on mice or other rodents. For obvious reasons, it would not be feasible to perform most neuroanatomical experiments on human tissue, but the question remains to what extent the acquired rodent data can be extrapolated to humans. Some parts of the mouse brain can be related to the human brain, e.g., the hippocampus, but other parts, like the neocortex, are less suitable for generalizations (Aboitiz et al., 2003; DeFelipe, 2010). This needs to be considered before extrapolating animal brain data to human brains.

Even before extrapolating mouse data to human brains, a number of other issues have to be addressed. Currently, connectome research is mainly focused on locating various cell types and determining their synaptic partners. Yook et al. (2013) argued that a connectome based on this information should really be named a “projectome” as it consists of only neurons and their projections. While acquiring more knowledge of the mouse projectome would certainly be useful, several authors have argued that we may come to understand brain network dynamics even better if we also assess synapse numbers, synapse types and synaptic strengths (O'Rourke et al., 2012; Yook et al., 2013; Markram et al., 2015). Research by Atasoy et al. (2014) has shown that there is indeed substantial variability in axonal release sites, even within one axonal segment. Also, Kasthuri et al. (2015) pointed out that information about spines and their closely apposed neurons is probably not sufficient for inferring synapses, as generally only one out of nine closely apposed neurons innervates a spine. Hence, future connectome research should probably not aim to reconstruct a “projectome” but rather to reconstruct a more ultrastructural “synaptome” (DeFelipe, 2010).

In 2013, Sporns reviewed a number of other factors that need to be taken into account for creating more realistic brain reconstructions. The first factor is individual structural variability, as no two animals will have the exact same connectome. The branching patterns of neurites and the placing and number of synapses can vary within a species, though there is still a remarkable level of functional stability (Sporns, 2013). In order to be able to make realistic reconstructions, we will need more knowledge about how this variability can arise while still maintaining similar brain function. Another factor is that of structural plasticity. Presently, various lines of evidence point toward continuous structural rearrangements of molecular and cellular components in the brain. Hence, we would be able to gain more knowledge about brain function and dysfunction if we could include structural plasticity in our models instead of just taking “snapshots” of brain anatomy (Sporns, 2013). Lastly, Sporns (2013) describes the issue of structure-function relations: how can brain function be inferred from brain structure? We still do not yet fully understand this type of relations, while this is a necessary condition for gathering information on brain function and dysfunction from brain anatomy. A dynamic interplay between neuroanatomy, neurophysiology and neuronal modeling research will be needed to further clarify these structure-function relations.

#### Data analysis: projection tracing and combining different types of data

While the previously mentioned issues concern the different types of data that are needed for biologically plausible brain mapping and modeling, the analysis of these various types of data is an issue too. As mentioned before, data analysis is currently the limiting step in connectome research as it is several orders of magnitude slower than data acquisition (Helmstaedter, 2013). Two important challenges in connectome data analysis are automation of projection tracing and combining large amounts of data from different types of experiments. As mentioned before, Bayesian modeling may provide a solution for data combination as Bayesian models allow the user to infer connectivity probabilities from information from different types of sources (Hinne et al., 2014, 2015). With regard to projection tracing, efforts are being made to not only increase automatic annotation speed and accuracy, but also to involve citizen annotators. For example, Kim et al. (2014) used the online community of *Eyewire* annotators to trace the projections of retinal cells. Data from human annotators can also be used to train segmentation algorithms, so combining automated projection tracing with human annotation might be a way increase data analysis speed and accuracy (Helmstaedter, 2013).

#### The complexity of neuroanatomical models

Just like there are unresolved issues regarding neuroanatomical data acquisition and analysis, there is ongoing discussion about neuroanatomical modeling. One specific issue is the complexity of brain models. When Markram et al. (2015) published their extensive and complex neocortical model, some researchers argued that we should not be making complex models for the sake of complexity, but that the models we create should be useful (Kupferschmidt, 2015). In 2009, James McClelland summarized his view on cognitive modeling in psychology as follows: “Models are not intended to capture fully the processes they attempt to elucidate. Rather, they are explorations of ideas about the nature of cognitive processes. In these explorations, simplification is essential—through simplification, the implications of the central ideas become more transparent.” This view can also be used when considering connectome modeling: connectome models can be used by scientists to test their ideas about structure-function relations in the brain.

Based on this point of view, we could also define how complex a connectome model should be. Since we want our ideas about structure-function relations in the brain to be biologically plausible, we need a biologically plausible model of the connectome to test our ideas. Hence, a connectome model should be complex enough to grasp our current knowledge about the biology of the brain. Currently, several researchers are working on brain models that use Peter's rule to infer synapses based on the distance between neurites (Shepherd et al., 2005; Stepanyants and Chklovskii, 2005; Hill et al., 2012). However, research performed by both Druckmann et al. (2014) and the neocortical reconstruction by Kasthuri et al. (2015) have indicated that axon-dendrite adjacency provides insufficient information to explain synapse formation. From this we are able to conclude that we need additional information to make our models more biologically plausible, and this in turn will enable us to improve our insight into structure-function relations in the brain.

As argued by McClelland (2009) and also in Kupferschmidt (2015), models are simplifications, so we should not aim to incorporate every single piece of information about the brain in our connectome models. The complexity of a model should be limited in the sense that when it sufficiently describes brain behavior, it should not be extended further. When a model does not yet sufficiently describe the behavior that it investigates, apparently there is information that we are missing. Tiesinga et al. (2015) indeed argued that building an integrated brain model will point us toward information about the brain that we are still missing but that is essential to understand brain behavior. Once we know this, an experimental approach could be defined to acquire this information.

To summarize, multiple strategies can and should be used in the field of connectomics. The integration of large amounts and different types of data is necessary to be able to create connectome models that are biologically plausible and will be able to inform scientists about structure-function relations in the brain. On the other hand, models are essentially simplifications that are created to help us put our ideas about brain structure and function to the test. Hence, the complexity of connectome models should not be increased further when a model already adequately describes the behavior that is being investigated. Thus, both data integration and simplification are relevant strategies for the field of connectomics. On what specific level of complexity data integration and simplification should converge is essentially dependent on the type of idea that needs to be tested with the model that is created.

## Author contributions

JLC performed literature research and created figures and tables; JLC, FC, and PHET wrote the paper.

### Conflict of interest statement

The authors declare that the research was conducted in the absence of any commercial or financial relationships that could be construed as a potential conflict of interest.
